# Prostatic Acid Phosphatase Is Required for the Antinociceptive Effects of Thiamine and Benfotiamine

**DOI:** 10.1371/journal.pone.0048562

**Published:** 2012-10-31

**Authors:** Julie K. Hurt, Jennifer L. Coleman, Brendan J. Fitzpatrick, Bonnie Taylor-Blake, Arlene S. Bridges, Pirkko Vihko, Mark J. Zylka

**Affiliations:** 1 Department of Cell and Molecular Physiology, UNC Neuroscience Center, University of North Carolina at Chapel Hill, Chapel Hill, United States of America; 2 Department of Clinical Medicine, Division of Clinical Chemistry, HUSLAB, University of Helsinki, Helsinki, Finland; The Hebrew University Medical School, Israel

## Abstract

Thiamine (Vitamin B1) is an essential vitamin that must be obtained from the diet for proper neurological function. At higher doses, thiamine and benfotiamine (S-benzoylthiamine O-monophosphate, BT)–a phosphorylated derivative of thiamine–have antinociceptive effects in animals and humans, although how these compounds inhibit pain is unknown. Here, we found that Prostatic acid phosphatase (PAP, ACPP) can dephosphorylate BT *in vitro*, in dorsal root ganglia (DRG) neurons and in primary-afferent axon terminals in the dorsal spinal cord. The dephosphorylated product S-benzoylthiamine (S-BT) then decomposes to O-benzoylthiamine (O-BT) and to thiamine in a pH-dependent manner, independent of additional enzymes. This unique reaction mechanism reveals that BT only requires a phosphatase for conversion to thiamine. However, we found that the antinociceptive effects of BT, thiamine monophosphate (TMP) and thiamine–a compound that is not phosphorylated–were entirely dependent on PAP at the spinal level. Moreover, pharmacokinetic studies with wild-type and *Pap^−/−^* mice revealed that PAP is not required for the conversion of BT to thiamine *in vivo*. Taken together, our study highlights an obligatory role for PAP in the antinociceptive effects of thiamine and phosphorylated thiamine analogs, and suggests a novel phosphatase-independent function for PAP.

## Introduction

In mammals, thiamine is an essential dietary supplement and is important for neurotransmission and neurological function [1], [2], [3], [4], [5]. Thiamine is absorbed in the intestine and transported from the extracellular space by thiamine transport receptors (THTR1 and THTR2, also called SLC19A2 and SLC19A3, respectively) [6], [7], [8]. Thiamine exists as the free molecule as well as in the form of several phosphate esters: thiamine monophosphate (TMP), thiamine diphosphate (TDP, also called thiamine pyrophosphate), and thiamine triphosphate (TTP) [9]. Inside cells, TDP is an important coenzyme in several biochemical processes, including carbohydrate and amino acid metabolism [10], [11], [12]. Additionally, several naturally occurring thiaminylated adenine nucleotides are found in bacteria and mammalian tissues [13], [14], [15]. Prokaryotes and eukaryotes thus contain a diverse repertoire of thiamine analogs.

In humans, thiamine deficiency causes Beriberi, a disease with neurological symptoms that include pain, neuropathy and memory loss. Thiamine deficiency is also associated with Wernicke-Korsakoff syndrome, Alzheimer’s disease, and diabetes [16], [17], [18], [19]. Thiamine deficiency can be treated by administering thiamine or compounds of the allithiamine class, the most commonly studied of which is BT [20], [21], [22], [23].

When administered at higher doses, thiamine and BT have antinociceptive effects in animal models of inflammatory pain and neuropathic pain [24], [25], [26], [27], [28], [29] and analgesic effects in humans, including patients with diabetic neuropathic pain [30], [31], [32]. Thiamine and BT are inexpensive and readily available over-the-counter, yet these compounds are not routinely used to treat chronic pain. *In vitro*, thiamine reduces nerve injury-induced hyperexcitability and modulates tetrodotoxin-resistant sodium currents in cultured small-to-medium diameter, presumably nociceptive, DRG neurons [25]. Currently, it is unknown what genes are required for the *in vivo* antinociceptive effects of thiamine and BT.

Recently, we found that the transmembrane isoform of PAP is expressed in small-to-medium diameter DRG neurons and dephosphorylates TMP extracellularly in these neurons and their spinal axon terminals [33], [34]. In light of these observations, we hypothesized that PAP might be required to dephosphorylate BT and mediate the antinociceptive effects of BT *in vivo*. Here, we tested this hypothesis using biochemical, genetic and pharmacokinetic approaches. Unexpectedly, we found that the antinociceptive effects of BT and thiamine–a compound that does not contain a phosphate group–were entirely dependent on PAP. Our findings suggest a novel, phosphatase independent, function for PAP in controlling the *in vivo* antinociceptive activity of thiamine and phosphorylated thiamine analogs.

**Figure 1:**
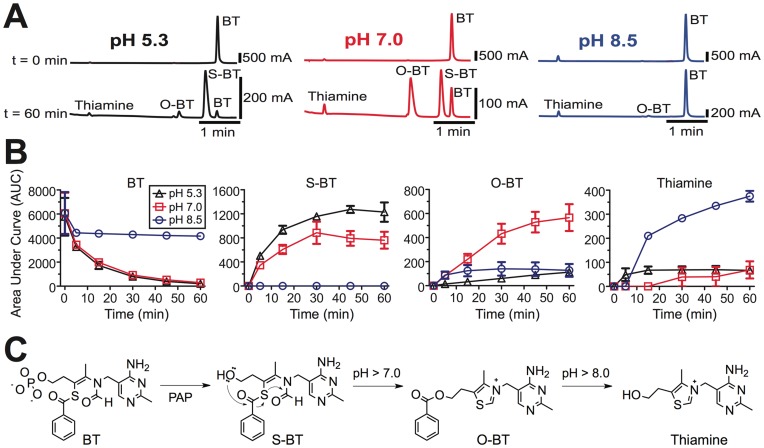
BT is dephosphorylated by PAP and then non-catalytically converted to thiamine. (**A,B**) LC-MS analysis of BT (3 mg/mL) and reaction products pre- and post-addition of hPAP (0.65 U) at acidic, neutral and alkaline pH values. Reactions were performed *in vitro* at 37°C for the indicated times. Only peaks with the expected mass values were integrated and analyzed. (**B**) Analyte peak areas for BT, intermediates (S-BT, O-BT) and thiamine. Reactions were performed in triplicate and the data are plotted as means ± s.e.m. (**C**) Reaction pathway. BT is stable at all pH values in the absence of PAP. Upon dephosphorylation, the intermediates (S-BT, O-BT) decompose to thiamine in a pH-dependent manner. These latter reactions proceed *in vitro* in the absence of enzymes (Fig. 2,3).

## Materials and Methods

### Ethics Statement

All procedures and behavioral experiments involving vertebrate animals were approved by the Institutional Animal Care and Use Committee at the University of North Carolina at Chapel Hill.

**Figure 2:**
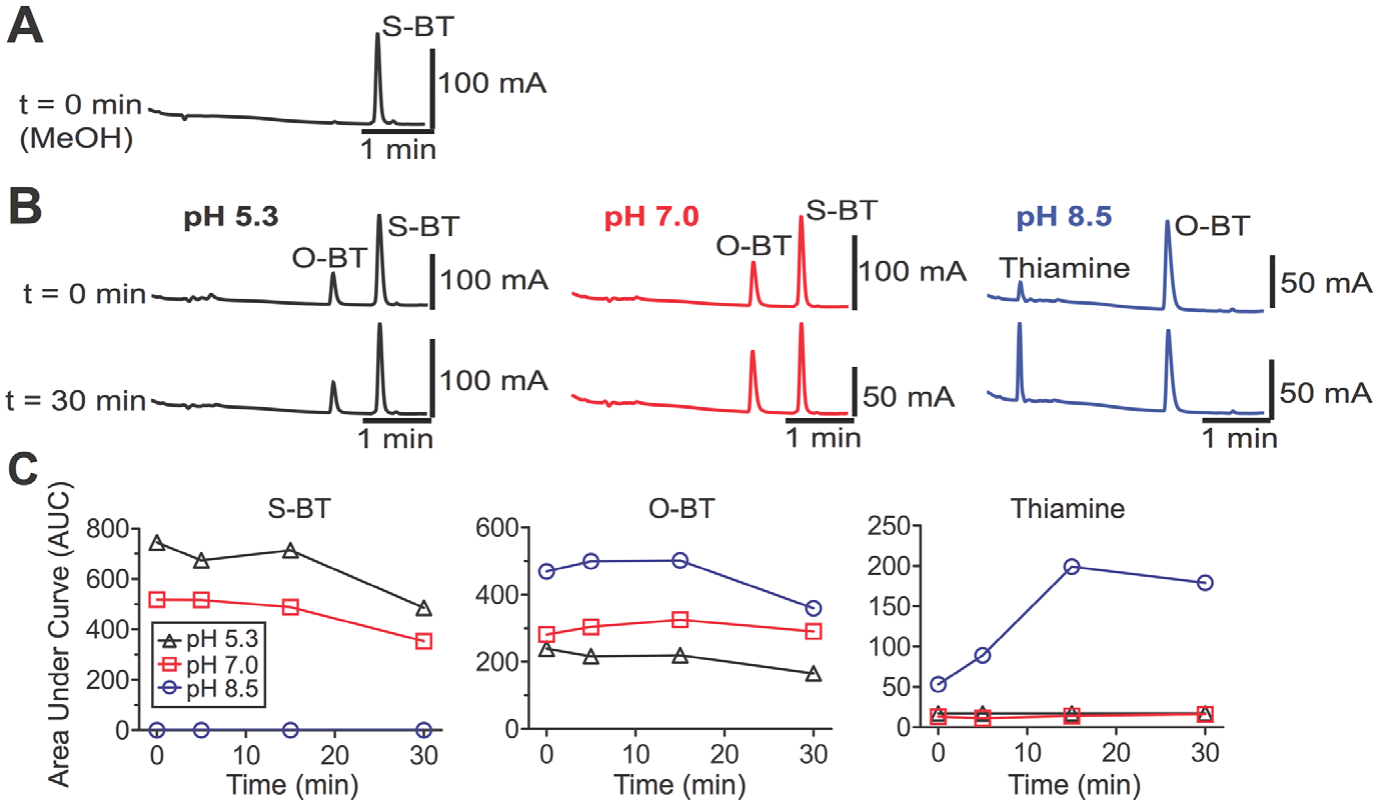
S-BT is non-enzymatically converted to O-BT and thiamine *in vitro*. (**A**) HPLC analysis of chromatographically purified S-BT (1 mg/mL) in methanol and (**B**) in aqueous solutions buffered at acidic, neutral and alkaline pH values (t = 0 and 30 min, respectively at 37°C). (**C**) Analyte peak areas for S-BT metabolites generated *in vitro*. Data are plotted as the total peak area observed at each time point.

**Figure 3:**
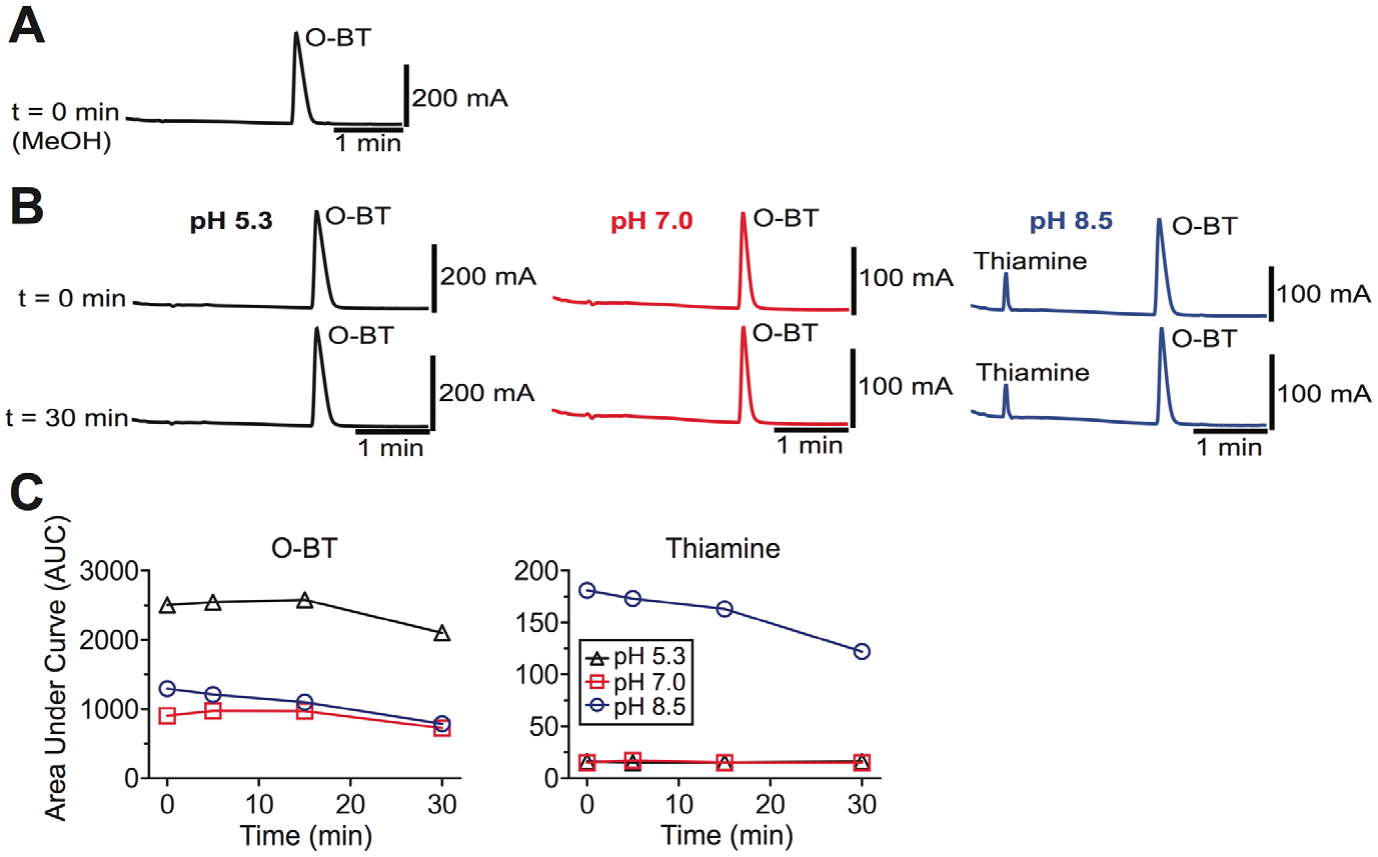
O-BT is non-enzymatically converted to thiamine *in vitro*. (**A**) HPLC analysis of chromatographically purified O-BT (1 mg/mL) in methanol and (**B**) in aqueous solutions buffered at acidic, neutral and alkaline pH values (t = 0 and 30 min, respectively at 37°C). (**C**) Analyte peak areas for O-BT and thiamine generated *in vitro*. Data are plotted as the total peak area observed at each time point.

### *In vitro* LC-MS Assay

Fresh BT (S-benzoylthiamine O-monophosphate, Sigma-Aldrich) was prepared in water prior to each experiment (100 mg/mL stock). Contrary to what has been reported [22], BT is soluble in water [35]. The phosphate group can be deprotonated to facilitate dissolution in water. We heated BT in a tightly-capped tube at 55°C and added 15 µL of 1 N NaOH every 10 min followed by vortexing until the solid was completely in solution. BT is susceptible to base hydrolysis if NaOH is added too quickly. BT hydrolysis can be further minimized by adding NaOH only after the solid is fully sedimented. The final pH was approximately 8.0. Prior to each experiment, we analyzed our stock BT solution by LC-MS to confirm that no hydrolysis products were present.

**Figure 4:**
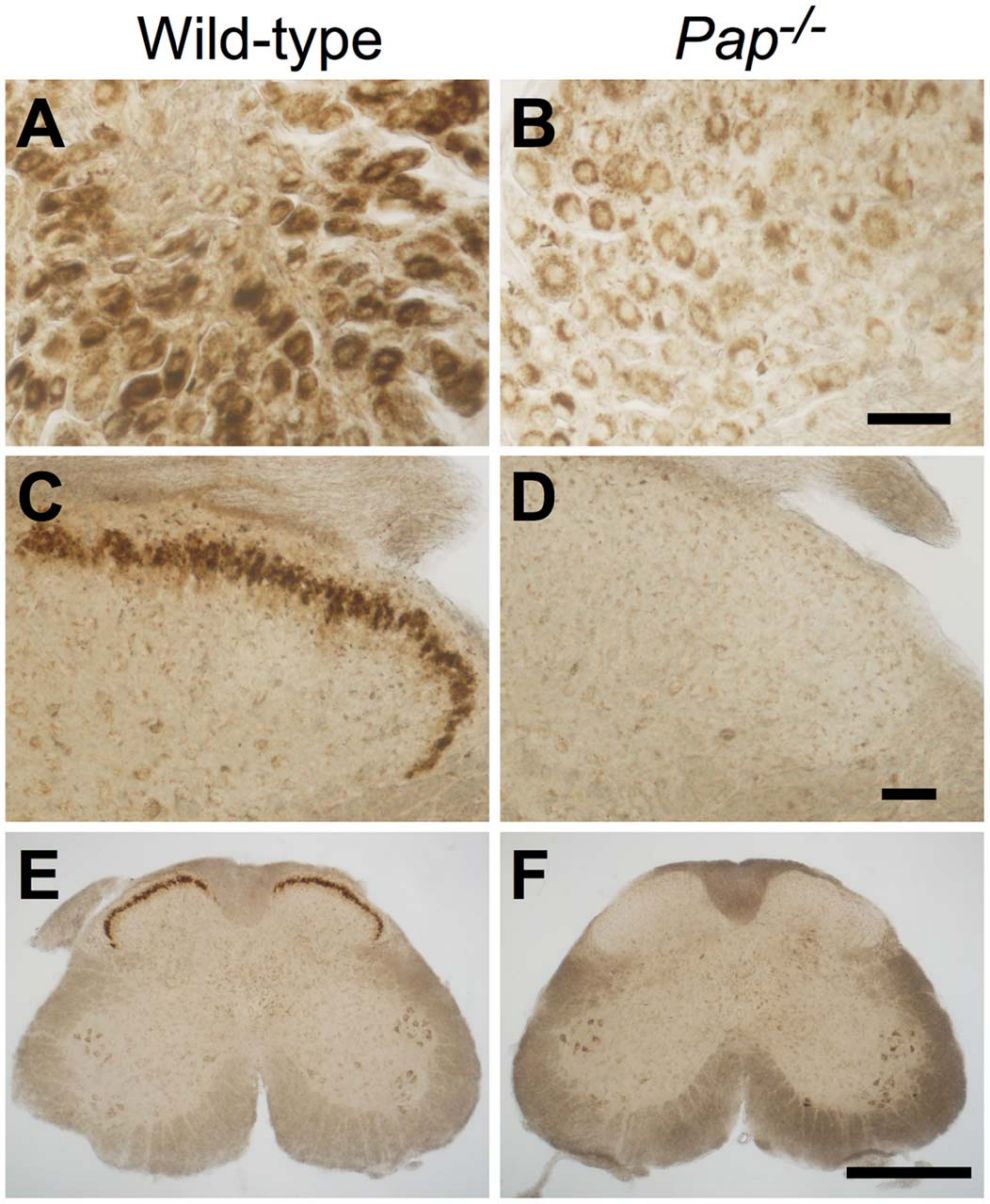
PAP dephosphorylates BT in small-to-medium diameter DRG neurons and afferent axon terminals in the spinal cord. (**A–F**) Sections from wild-type and *Pap^−/−^* mouse (**A,B**) DRG and (**C–F**) spinal cord stained using enzyme histochemistry (6 mM BT at pH 7.0). Scale bar, 50 µm in (**B,D**), 500 µm in (**F**).

The reaction of BT with hPAP was monitored *in vitro* using LC-MS. Samples containing BT (15 mg/mL, 1100 µL total volume) were diluted in one of three different buffers: 100 mM sodium acetate, pH 5.3; 100 mM HEPES, pH 7.0; or 100 mM Tris, pH 8.5. Samples were incubated at 37°C and the reaction was initiated with the addition of hPAP (3.25 U diluted in 0.9% saline, Sigma-Aldrich). Aliquots (200 µL) were removed to a fresh microcentrifuge tube at 0, 5, 15, 30, 45 and 60 min. Reactions were terminated by adding methanol (480 µL) and chloroform (160 µL) to precipitate the protein, followed by addition of water (640 µL) and vortex mixing. After centrifugation (2,152 x g for 5 min at room temperature), the aqueous methanol-water phase was transferred to a separate microcentrifuge tube for analysis.

**Figure 5:**
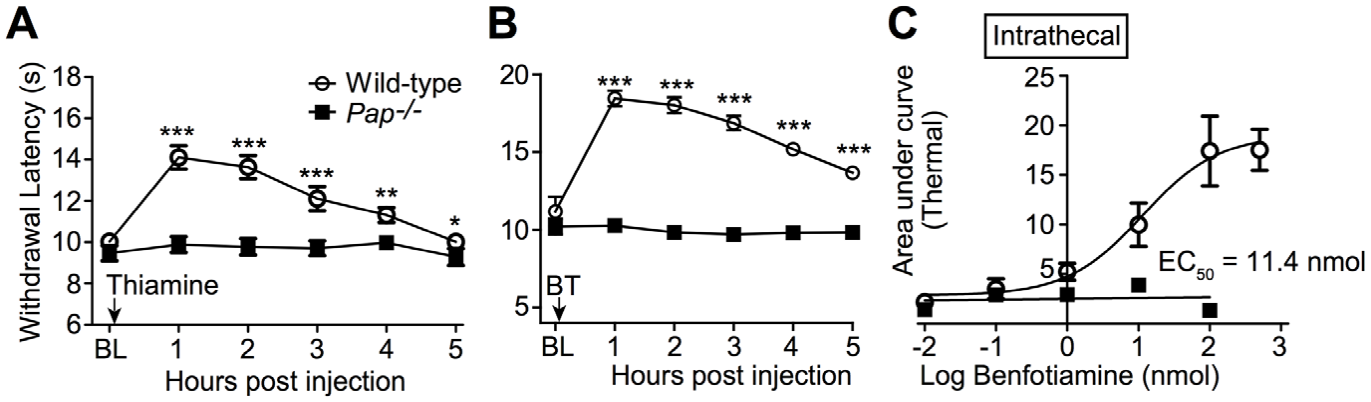
The thermal antinociceptive effects of thiamine and BT, when delivered spinally, are entirely dependent on PAP. (**A,B**) Wild-type and *Pap^−/−^* male mice were tested for noxious thermal sensitivity (hindpaw, radiant heating) before (baseline, BL) and following i.t. injection of (**A**) 50 nmol thiamine (n = 30 mice/group) or (**B**) 100 nmol BT (n = 20 mice/group). Paired t tests were used to compare responses at each time point to BL responses. *P<0.05, **P<0.005, ***P<0.0005. (**C**) BT (i.t.) dose-response. Thermal sensitivity monitored for 5 hr after injecting BT i.t. (0.01–100 nmol). Plotted as area under the curve. n = 10 mice per group. Data were fit by non-linear regression to a sigmoidal dose response equation, and the EC_50_ value was calculated in GraphPad Prism. Data plotted as means ± s.e.m.

LC-MS analysis was performed at room temperature on an Agilent ZORBAX Eclipse Plus C18 Rapid Resolution HT column (4.6 x 50 mm, 1.8 µm). Mobile phase A was water and mobile phase B was methanol, which were both supplemented with 0.1% acetic acid. Samples were run with the following gradient (1 mL/min flow rate, 7 min total run time): 10%–100% B in 5 min, hold at 100% B for 2 min, and return to starting conditions in a 1 min post-run. Sample injection volume was 5 µL. Peak area was quantified using the Agilent ChemStation software. Only peaks associated with the predicted mass for a given BT metabolite were quantified.

**Figure 6:**
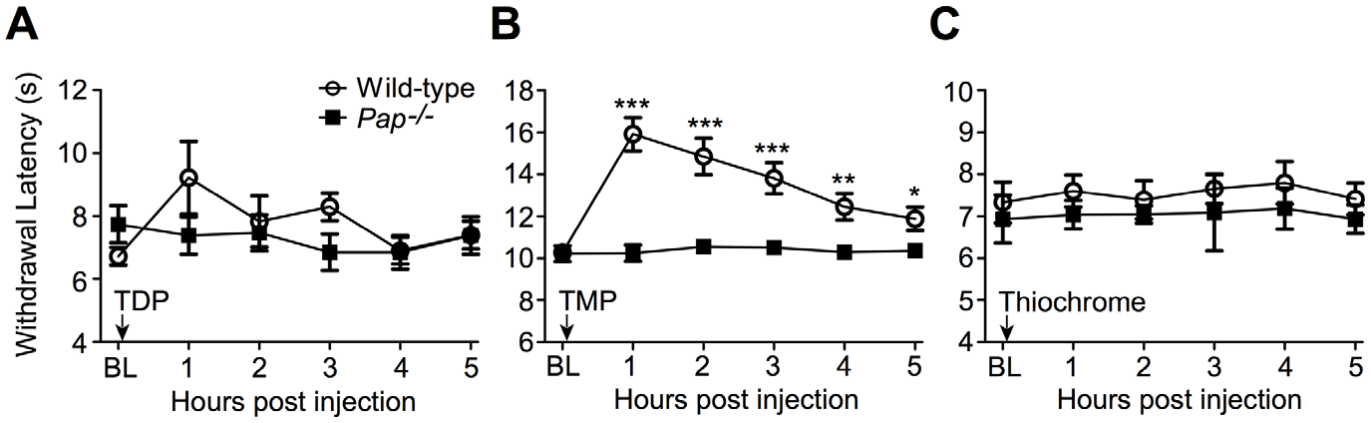
TMP, but not other thiamine metabolites, inhibits noxious thermal sensitivity in a PAP-dependent manner. (**A–C**) Wild-type and *Pap^−/−^* male mice were tested for noxious thermal sensitivity (hindpaw, radiant heating) before (baseline, BL) and following i.t. injection of (**A**) 25 nmol TDP (n = 10 wild-type mice, n = 9 *Pap^−/−^* mice), (**B**) 100 nmol TMP (n = 17 wild-type mice, n = 18 *Pap^−/−^* mice) and (**C**) 25 nmol thiochrome (n = 10 wild-type mice, n = 9 *Pap^−/−^* mice). (**A–C**) T tests were used to compare responses at each time point to baseline (BL). *P<0.05, **P<0.005, ***P<0.0005. Data are plotted as means ± s.e.m.

### Animals

All experiments were performed as previously described with male mice during the light phase, raised under a 12∶12 light:dark cycle [33]. C57BL/6 mice (2–4 months in age) were purchased from Jackson Laboratories, and *Pap^−/−^* and *A_1_R^−/−^* mice were backcrossed to C57BL/6J mice for 12 generations. *A_1_R^−/−^, Pap^−/−^* double knockout mice were generated by breeding backcrossed *A_1_R^−/−^* and *Pap^−/−^* mice.

**Figure 7:**
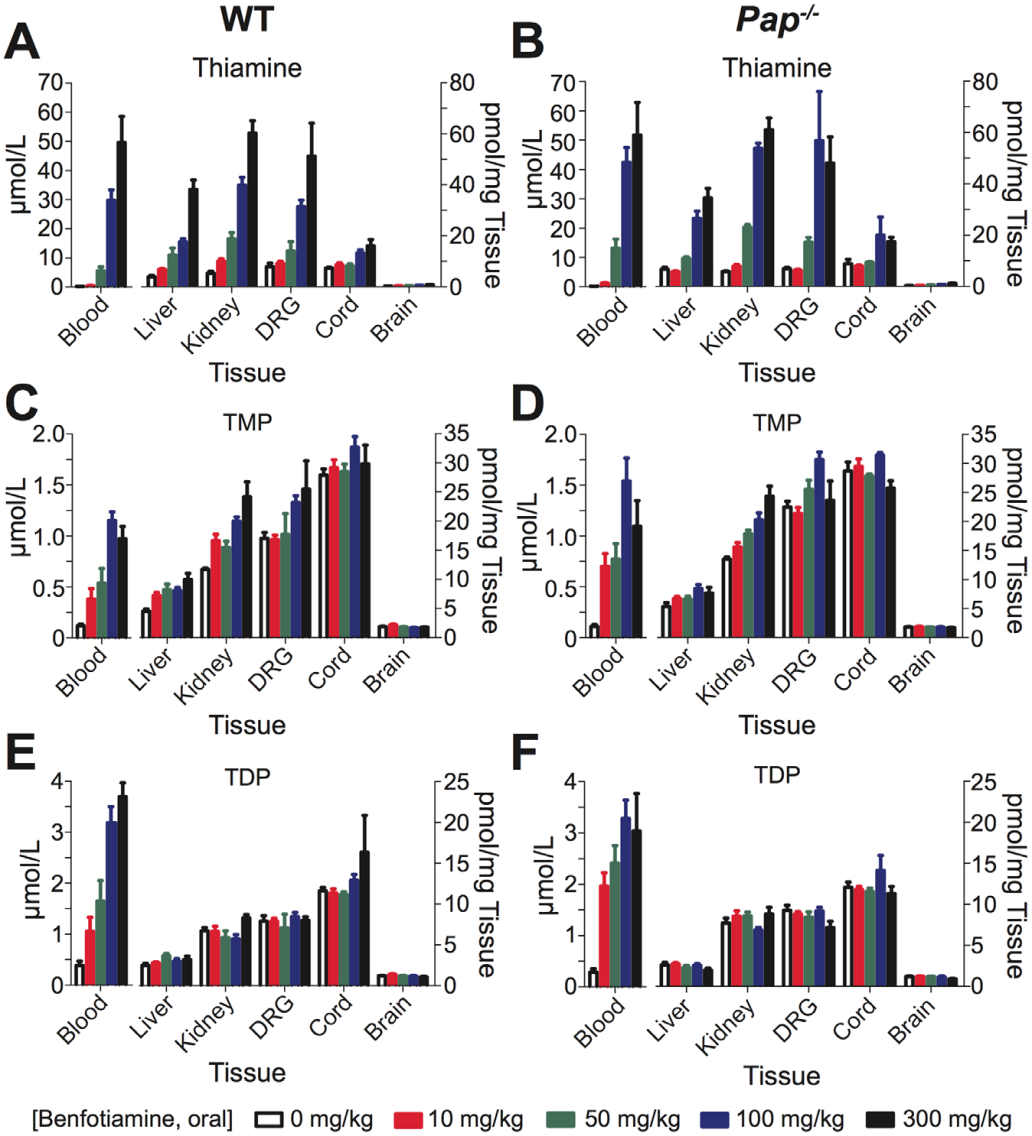
Pharmacokinetic profiles of thiamine, TMP and TDP do not differ between wild-type and *Pap^−/−^* mice following oral administration of BT. (**A–F**) BT (0–300 mg/kg, p.o.) was administered to wild-type and *Pap^−/−^* mice (n = 5/dose). The indicated tissues were harvested 1 h post oral gavage then were homogenized in 10% trichloroacetic acid. Fluorescent thiochrome derivatives were monitored by reversed phase HPLC to detect (**A–B**) thiamine, (**C–D**) TMP, and (**E–F**) TDP. Peak area values were converted to units of concentration for each thiamine derivative and normalized by the weight of tissue in each replicate. There were no significant differences between wild-type and *Pap^−/−^* mice. Data are plotted as means ± s.e.m.

### Drugs and Injections

Intrathecal injections (5 µL) were performed using acute lumbar puncture without anesthesia [36]. BT (from stock, made as described above), thiamine monophosphate hydrochloride (TMP), thiamine hydrochloride, thiamine pyrophosphate (TDP) and thiochrome were purchased from Sigma-Aldrich and diluted in 0.9% saline. Prior to injection, the purity of the final BT solution was monitored by LC-MS as described above. A metal gavage needle fitted to a syringe was used to deliver vehicle/BT p.o.

**Figure 8:**
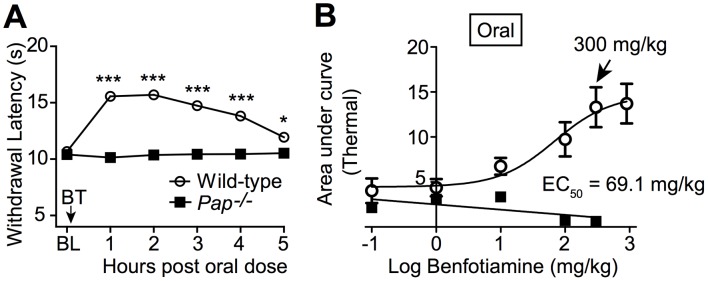
The thermal antinociceptive effect of BT, when delivered orally, is entirely dependent on PAP. (**A**) Wild-type (n = 21) and *Pap^−/−^* (n = 15) male mice were tested for noxious thermal sensitivity (hindpaw, radiant heating) before (BL) and following oral administration of BT (300 mg/kg, p.o.). Paired t tests were used to compare responses at each time point to BL responses. *P<0.05, ***P<0.0005. (**B**) BT (p.o.) dose-response. Thermal sensitivity monitored for 5 hr after administering BT orally (0.1–300 mg/kg). Plotted as area under the curve. n = 10 male mice per group. The data were fit by non-linear regression to a sigmoidal dose response equation, and the EC_50_ value was calculated in GraphPad Prism. Data are plotted as means ± s.e.m.

### Behavior

All mice were acclimated to the experimenter, the room and the experimental apparatus for 3–5 days prior to behavioral testing. Thermal sensitivity was monitored using the Hargreaves method, where the radiant heat source was calibrated to elicit a paw withdrawal reflex of approximately 10 s in naïve mice (cutoff time of 20 s). Complete Freund’s adjuvant (20 µL, MP Biomedicals) was injected under the glabrous skin to inflame one hindpaw. Spared nerve injury (SNI) was used to model neuropathic pain [33], [37].

**Figure 9:**
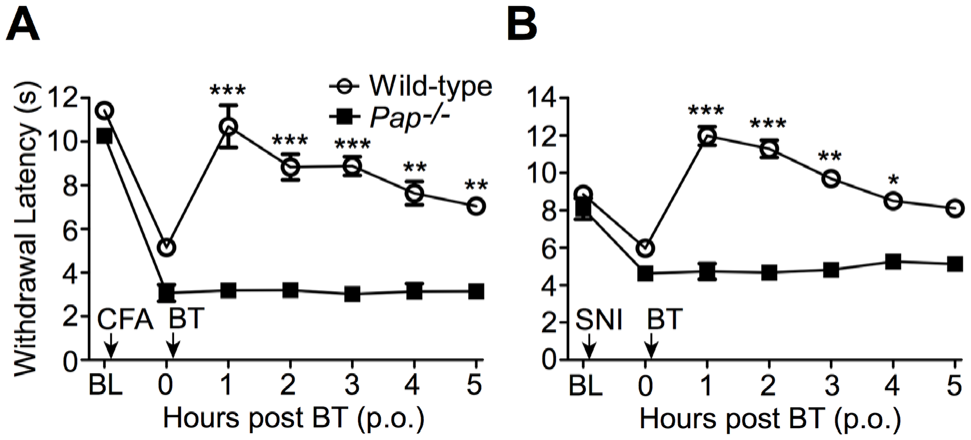
BT has thermal antihyperalgesic effects in chronic inflammatory and neuropathic pain models that are entirely PAP-dependent. (**A**) Inflammatory pain model. CFA was injected into the hindpaw of wild-type (n = 20) and *Pap^−/−^* (n = 9) male mice, then BT (300 mg/kg, p.o.) was administered 1 day later. Thermal sensitivity monitored at the indicated times in the inflamed hindpaw. (**B**) Neuropathic pain model. The sural and common peroneal branches of the sciatic nerve were ligated and then transected in wild-type (n = 28) and *Pap^−/−^* (n = 20) male mice. BT (300 mg/kg, p.o.) was administered 2 days later. Thermal sensitivity monitored at the indicated times in the nerve-injured hindpaw. T tests were used to compare responses between wild-type and *Pap^−/−^* mice. *P<0.05, **P<0.005, ***P<0.0005. Data are plotted as means ± s.e.m.

### Tissue Collection

Vehicle (0.9% saline) or BT (10–300 mg/kg) was administered to adult male wild-type and *Pap^−/−^* mice (2–4 months in age) by oral gavage (100 µL/mouse). One hour post-treatment, mice were given a lethal dosage of pentobarbital (50 mg/kg, i.p.). The chest cavity was opened and blood was withdrawn by direct cardiac puncture from the right atrium using a sterile 1 mL syringe with a 23 gauge, ¾ inch needle (BD Biosciences). The needle was removed, and the blood was immediately added to a tube containing 250 I.U. heparin sodium salt (Sigma-Aldrich). The liver, kidneys, brain, DRG and spinal cord were removed and flash frozen on dry ice. All samples were stored at −80°C prior to homogenization.

**Figure 10:**
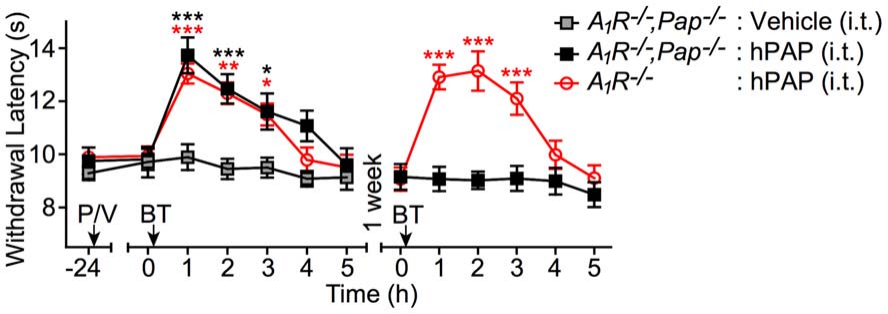
Rescue experiment reveals that PAP is exclusively required at the spinal level to mediate the antinociceptive effects of systemic BT. hPAP (P; 250 mU) was injected i.t. into *A_1_R^−/−^,Pap^−/−^* (n = 10) and *A_1_R^−/−^* (n = 10) male mice. Vehicle (V; 0.9% saline) was injected into a separate group of *A_1_R^−/−^, Pap^−/−^* (n = 10) male mice. BT (300 mg/kg, p.o.) was administered 24 h later. One week later, BT (300 mg/kg, p.o.) was again administered to the mice that were previously injected with hPAP. Noxious thermal sensitivity in the hindpaw was measured at the indicated times. Paired t tests were used to compare responses at each time point to the response before administration of BT. *P<0.05, **P<0.005, ***P<0.0005. Data are plotted as means ± s.e.m.

### Thiochrome Detection by HPLC

Samples were prepared in pre-tared screw-cap centrifuge tubes with 6 zirconium oxide beads (1.4 mm, Bertin Technologies) and 3–5 volumes 10% trichloroacetic acid depending on tissue weight. In addition, a single 2.8 mm zirconium oxide bead was added to the kidney samples to aid in homogenization. Tissues were homogenized in the Precellys 24 (Bertin Technologies) at 4°C (5,000 x g, 25 s, 3 cycles). Samples were incubated on ice for 15 min. and the protein and insoluble fraction was harvested by centrifugation (13,000 x g for 6 min). The aqueous phase was extracted with 2 x 2 mL water-saturated methyl *t*-butyl ether (MTBE) in glass test tubes. Thiamine metabolites were oxidized to thiochrome derivatives with the addition of potassium ferric cyanide (0.6 mM in 15% NaOH). Sample preparation and all chromatographic steps were performed as previously described [38].

### Histology

Enzyme histochemistry was performed as described previously [33]. Briefly, tissue sections were washed twice with 40 mM Trizma-Maleate (TM) buffer, pH 7.0, and once with TM buffer containing 8% (w/v) sucrose. The tissue was then incubated for 2 hr and 37°C in TM buffer with 8% sucrose (w/v), 6 mM BT, and 2.4 mM lead nitrate (made fresh prior to use). Samples were washed with 2% acetic acid for 1 min, and three times with TM buffer. Samples were treated with 1% sodium sulfide for 10 sec and washed several times with phosphate-buffered saline (PBS), pH 7.4.

## Results

### BT is a PAP Substrate and Decomposes to Thiamine Upon Dephosphorylation

To determine if PAP could dephosphorylate BT *in vitro*, we incubated purified human (h)PAP protein (the secretory isoform) with BT then monitored reaction products over time by liquid chromatography mass spectrometry (LC-MS). We found that hPAP dephosphorylated BT to S-BT at an acidic pH and neutral pH but S-BT was not detected at an alkaline pH (Fig. 1A,B). Peaks for O-BT and thiamine appeared at all pH values, with O-BT production favored at pH 7.0, and thiamine production favored at pH 8.5. The structures of all compounds are shown in Fig. 1C.

It was previously assumed that S-BT was converted to thiamine via a thioesterase-dependent mechanism [39]. However, no thioesterases were present in our *in vitro* reactions, suggesting thiamine was produced via an alternative reaction mechanism. To determine if thiamine could be generated in a non-enzymatic fashion from S-BT and O-BT, we purified the S-BT and O-BT reaction intermediates by high-performance liquid chromatography (HPLC; Fig. 2A and 3A, respectively), then monitored the stability of these compounds in the absence of enzymes. Intriguingly, we found that S-BT underwent an intramolecular rearrangement to O-BT at pH 5.3 and pH 7.0, then partially decomposed to thiamine at pH 8.5 (Fig. 2B-C). Likewise, purified O-BT was stable at pH 5.3 and pH 7.0 then decomposed to thiamine at pH 8.5 (Fig. 1C, 3B-C). Taken together, our data reveals that BT is stable *in vitro* in the absence of enzymes. However, once BT is dephosphorylated at physiological pH, S-BT, O-BT and thiamine are produced (Fig. 1C). BT thus only requires a phosphatase for conversion to thiamine.

### PAP Dephosphorylates BT in Nociceptive Circuits

We previously found that TMP is a PAP-specific substrate in nociceptive neurons [33]. To determine if PAP could also dephosphorylate BT in small-to-medium diameter, presumably nociceptive neurons, we performed BT enzyme histochemistry with sections of DRG and spinal cord from wild-type and *Pap^−/−^* mice. We found that small-to-medium diameter neurons and their axon terminals in lamina II of the spinal cord were intensely stained at pH 7.0 in wild-type mice (Fig. 4A,C,E) but were not intensely stained in *Pap^−/−^* mice (Fig. 4B,D,F). Thus PAP can dephosphorylate BT extracellularly in DRG neurons and in dorsal spinal cord.

### The Antinociceptive Effects of BT, TMP and Thiamine are PAP-dependent

Since PAP can dephosphorylate BT *in vitro* and in nociceptive neurons, we next hypothesized that PAP might be required for the antinociceptive effects of BT but not thiamine. To test this hypothesis, we intrathecally (i.t.) injected wild-type and *Pap^−/−^* mice with these compounds, then monitored noxious thermal sensitivity in the hindpaw. Consistent with previous studies [24], [25], [29], we found that thiamine (50 nmol, Fig. 5A) and BT (100 nmol, Fig. 5B) increased paw withdrawal latency for 5 hr, highlighting a thermal antinociceptive effect for these compounds. The antinociceptive effect of BT was dose-dependent in wild-type mice (EC_50_ = 11.4 nmol, Fig. 5C). Strikingly, the antinociceptive activity of each compound was completely absent in the *Pap^−/−^* mice (Fig. 5A-C). The lack of activity following BT administration was predicted; however, the lack of activity following thiamine administration–a compound that is not phosphorylated–was unexpected. These data unexpectedly revealed that PAP was required for the biological activity of BT and thiamine, suggesting a phosphatase-independent function for PAP *in vivo*.

Since thiamine exists in a variety of forms *in vivo*, we postulated that PAP might instead elicit antinociceptive effects by generating an alternate thiamine metabolite. If this were the case, we could bypass a requirement for PAP by injecting this putative metabolite, and show it has PAP-independent antinociceptive effects. Thus, to test this hypothesis, we studied the antinociceptive properties of several commercially-available thiamine derivatives: two phosphorylated metabolites (TMP and TDP) and the oxidation product of thiamine (thiochrome) (Figure 6). Following i.t. administration, we found that neither TDP (25 nmol) nor thiochrome (25 nmol) had antinociceptive effects in wild-type or *Pap^−/−^* mice (Fig. 6A and C, respectively). In contrast, the PAP-specific substrate, TMP (100 nmol), was active in wild-type mice but not *Pap^−/−^* mice (Fig. 6B). Since none of these metabolites acted independent of PAP, none of these compounds are likely to be an active, PAP-independent, metabolite *in vivo*.

### Pharmacokinetic Profile of Oral BT in Wild-type and *Pap^−/−^* Mice

Given that BT had no antinociceptive effects in *Pap^−/−^* mice, we next sought to determine if *Pap^−/−^* mice were unable to absorb or metabolize BT when administered orally. To address this, we administered a range of BT doses (0–300 mg/kg, p.o.) to wild-type and *Pap^−/−^* mice, sacrificed the mice 1 hr later, and then collected six different tissues for analysis (Fig. 7). Several metabolites of BT (thiamine, TMP, TDP) can be monitored in tissue homogenates by HPLC after conversion to fluorescent thiochrome derivatives [9], [22], [38]. In wild-type and *Pap^−/−^* mice, we found that thiamine concentration increased in a dose-dependent manner in each tissue studied, with the largest increases in concentration observed in peripheral tissues (blood, liver, kidney, and DRG) (Fig. 7A,B). This included a small but significant dose-dependent increase of thiamine concentration in the brain (2.3 fold increase at 300 mg/kg BT dose), consistent with previous studies [18]. We also observed a dose-dependent increase in TMP in the blood and peripheral tissues of wild-type and *Pap^−/−^* mice (Fig. 7C,D). TDP levels were dose-dependently elevated only in the blood of both strains (Fig. 7E,F). Importantly, there were no significant differences in thiamine, TMP or TDP concentration between wild-type and *Pap^−/−^* mice at any dose or in any tissue. Wild-type and *Pap^−/−^* mice are thus equally capable of absorbing and metabolizing BT to thiamine, TMP and TDP. This data suggest that other phosphatases, such as alkaline phosphatases [22], [39], metabolize BT to thiamine *in vivo*.

### Antinociceptive Effects of BT Require PAP at the Spinal Level

Though we were unable to resolve precisely why PAP was required for the antinociceptive effects of BT *in vivo*, we next sought to determine if BT had PAP-dependent antinociceptive effects when delivered systemically via a therapeutically relevant route, namely orally (via oral gavage; p.o.). It has been demonstrated previously that BT is more readily absorbed from the intestine than thiamine [22], [23], [39]. Following oral delivery, we found that BT (p.o.) had thermal antinociceptive effects in wild-type mice but not *Pap^−/−^* mice at all doses tested (Fig. 8A,B, with EC_50_ = 69.1 mg/kg in wild-type mice). These data and our data above collectively reveal that PAP is required for the antinociceptive effects of BT when delivered spinally or systemically.

BT also has thermal antihyperalgesic effects in models of chronic pain [28]. To determine if these thermal antihyperalgesic effects were PAP-dependent, we administered BT (p.o.) to wild-type and *Pap^−/−^* mice after inflaming one hindpaw with Complete Freund’s adjuvant (CFA; a model of inflammatory pain; Fig. 9A). We also evaluated BT in the spared nerve injury (SNI) model of neuropathic pain (Fig. 9B). In both chronic pain models, BT (300 mg/kg, p.o.) inhibited thermal hypersensitivity in the inflamed/injured paw of wild-type mice but had no effect in *Pap^−/−^* mice, thus revealing a requirement for PAP in the thermal antihyperalgesic effects of systemic BT.

BT reportedly has mechanical antiallodynic effects in models of neuropathic pain [28]. Although, others found that the BT metabolite thiamine does not have antiallodynic effects [25], [29]. Consistent with these latter studies, we found that BT (300 mg/kg, p.o.) did not have mechanical antiallodynic effects in wild-type or *Pap^−/−^* mice following CFA-induced inflammation or SNI surgery (data not shown, using an electronic von Frey apparatus to monitor mechanical allodynia).

PAP is expressed in nociceptive neurons in DRG as well as other tissues of the body [33], [40]. To determine if the antinociceptive effects of systemic BT were dependent on PAP at the spinal level, we next performed a biochemical rescue experiment with *Pap^−/−^* mice. This experiment was based on our observation that hPAP had A_1_R-dependent antinociceptive effects that lasted 3 days when injected i.t. [33]. We reasoned that we could inject hPAP (250 mU, i.t.) into *Pap^−/−^* mice and restore PAP activity at the spinal level in an otherwise *Pap*-deficient background. As part of this experiment, we generated *Pap^−/−^, A_1_R^−/−^* double knockout mice, to prevent the A_1_R-dependent antinociceptive effects of hPAP [33] from obscuring the antinociceptive effects of BT.

We then monitored noxious thermal sensitivity in *Pap^−/−^, A_1_R^−/−^* mice before and after injecting vehicle or hPAP (i.t.) (Fig. 10). In parallel, *A_1_R^−/−^* mice were injected with hPAP (i.t.) as a control. One day later, we administered BT (300 mg/kg, p.o.) systemically to all the mice. Remarkably, systemic BT inhibited thermal nociception in *Pap^−/−^, A_1_R^−/−^* mice that received hPAP (i.t.) but had no effect in *Pap^−/−^, A_1_R^−/−^* mice that received vehicle (i.t.) (Fig. 10). We then administered BT (p.o.) to these same hPAP injected mice one week later, a time when spinal hPAP is no longer active [33]. This second BT (p.o.) dose was no longer effective in *Pap^−/−^, A_1_R^−/−^* mice (Fig. 8, black squares) but remained effective in the *A_1_R^−/−^* controls (Fig. 10, red line). Taken together, this biochemical rescue experiment reveals that the antinociceptive effects of systemic BT administration were entirely due to PAP activity at the spinal level. Additionally, since BT was active in *A_1_R^−/−^* mice, this experiment indicates that BT and its metabolites do not inhibit nociception via A_1_R.

## Discussion

At low doses, thiamine acts as a vitamin and is essential for cellular and neurological functions. At higher doses, thiamine and BT have antinociceptive effects in animal models of inflammatory pain and neuropathic pain [24], [25], [26], [28], [29]. Thiamine and BT also have analgesic effects in humans, including patients with diabetic neuropathic pain [30], [31], [32], though the analgesic effects in these human studies are admittedly modest. More clinical studies are certainly needed to assess safety and efficacy of BT [23], [41]. In addition, little is known about how thiamine and BT inhibit nociception at a mechanistic level. This lack of clinical and preclinical knowledge likely limits the use of these inexpensive and readily-available (over the counter) compounds to treat some forms of chronic pain.

Previously, we found that PAP could dephosphorylate extracellular TMP in DRG and the spinal cord of mice [33], raising the possibility that PAP might dephosphorylate the related analog BT to thiamine, and hence mediate the antinociceptive properties of BT. We directly tested this possibility by assessing the extent to which PAP dephosphorylates BT *in vitro and vivo* and by studying the importance of PAP in BT-mediated antinociception.

We found that BT is a substrate for PAP *in vitro* and that BT histochemical staining was eliminated in *Pap^−/−^* mouse tissue. The latter experiments provide genetic support that PAP dephosphorylates BT in nociceptive neurons and afferent terminals in the spinal dorsal horn. The necessity of PAP was further demonstrated *in vivo*, as the antinociceptive effects of BT were eliminated in *Pap^−/−^* mice but could be rescued following spinal administration of PAP protein.

These data initially suggested that PAP might be required to convert BT to thiamine *in vivo*. Since thiamine does not contain a phosphate group, we predicted thiamine would have antinociceptive effects independent of PAP. However, to our surprise, we found that the antinociceptive effects of thiamine were also dependent on PAP, suggesting that PAP might have other, non-phosphatase-related functions *in vivo*.

For example, PAP might transphosphorylate thiamine to TMP or TDP. Previous studies have shown that alkaline phosphatases and acid phosphatases, including PAP, can transphosphorylate substrates in the presence of phosphate donor compounds [42], [43], [44], [45]. In principle, this transphosphorylation activity could be bypassed *in vivo* by injecting TMP or TDP directly into *Pap^−/−^* mice. However, we found that TDP (i.t.) had no antinociceptive effects in wild-type or *Pap^−/−^* mice (Fig. 6A), and TMP (i.t.) was active only in wild-type mice (Fig. 6B). Since neither TDP nor TMP had PAP-independent antinociceptive effects, we conclude that these metabolites are not downstream of PAP and do not serve as the active compounds. Instead, the antinociceptive effects of TMP also require PAP, placing TMP upstream of PAP in this biochemical pathway.

Alternatively, we considered the possibility that PAP might directly or indirectly oxidize thiamine. Thiamine is oxidized to thiochrome *in vivo* [46], and thiochrome can positively modulate a class of muscarinic acetylcholine receptors that inhibit nociception [47], [48], [49]. Following direct spinal administration, we found that thiochrome had no antinociceptive properties in wild-type or *Pap^−/−^* mice (Fig. 6C), making it unlikely that the antinociceptive effects of BT, TMP or thiamine occur via metabolism and oxidation to thiochrome.

Lastly, if PAP functioned *in vivo* as a phosphatase for BT or TMP, we would predict a buildup of one or more phosphorylated metabolites (i.e., TMP) coupled with a subsequent decrease in another metabolite (i.e., thiamine) in the *Pap^−/−^* mice. However, the pharmacokinetic profiles revealed no difference in thiamine, TMP or TDP levels between the two strains. Since all of these BT metabolites were present at normal levels in *Pap^−/−^* mice, our data suggests that other enzymes, such as alkaline phosphatases [39], facilitate absorption and metabolism of BT.

Further studies will be needed to determine precisely why PAP is required for the antinociceptive effects of BT, TMP and thiamine. Our data support a number of possibilities, including that a) PAP might transform thiamine into an active compound via a novel catalytic activity, b) PAP could serve as a thiamine/thiamine analog receptor or be an essential subunit of such a receptor, or c) PAP could facilitate binding of thiamine or thiamine analogs to a receptor. Indeed, the antinociceptive effects of thiamine and BT are maximal within the first hour post-delivery, suggesting that all of these compounds act via a receptor-dependent mechanism. BT can also inhibit biochemical pathways implicated in hyperglycemic damage [27], [31], [50], although inhibition of these pathways was detected after 6 hr, well after the rapid-onset antinociceptive effects of BT.

In summary, our study reveals an essential and unexpected role for PAP in mediating the antinociceptive effects of BT, TMP and thiamine. This function is distinct from the ectonucleotidase activity of PAP [33]. Our study also suggests a novel *in vivo* function for PAP that is unrelated to its phosphatase activity. Our mechanistic findings will encourage further research into BT and analogs and suggest a broader role for PAP in regulating thiamine-dependent processes throughout the body [40].

## References

[1] Enomoto KI, Edwards C (1985) Thiamine blockade of neuromuscular transmission. Brain Research 358: 316–323. 2416387 10.1016/0006-8993(85)90976-x

[2] Bettendorff L (1994) Thiamine in excitable tissues - Reflections of a non-cofactor role. Metabolic Brain Disease 9: 183–209. 7838063 10.1007/BF01991194

[3] Ba A (2008) Metabolic and Structural Role of Thiamine in Nervous Tissues. Cellular and Molecular Neurobiology 28: 923–931. 18642074 10.1007/s10571-008-9297-7 PMC11514992

[4] Henriquez-Sanchez P, Sanchez-Villegas A, Doreste-Alonso J, Ortiz-Andrellucchi A, Pfrimer K, et al (2009) Dietary assessment methods for micronutrient intake: a systematic review on vitamins. British Journal of Nutrition 102: S10–S37. 20100364 10.1017/S0007114509993126

[5] Batifoulier F, Verny MA, Chanliaud E, Remesy C, Demigne C (2006) Variability of B vitamin concentrations in wheat grain, milling fractions and bread products. European Journal of Agronomy 25: 163–169.

[6] Said HM, Ortiz A, Subramanian VS, Neufeld EJ, Moyer MP, et al (2001) Mechanism of thiamine uptake by human colonocytes: studies with cultured colonic epithelial cell line NCM460. American Journal of Physiology-Gastrointestinal and Liver Physiology 281: G144–G150. 11408266 10.1152/ajpgi.2001.281.1.G144

[7] Rindi G, Laforenza U (2000) Thiamine intestinal transport and related issues: Recent aspects. Proceedings of the Society for Experimental Biology and Medicine 224: 246–255. 10964259 10.1046/j.1525-1373.2000.22428.x

[8] Ganapathy V, Smith SB, Prasad PD (2004) SLC19: the folate/thiamine transporter family. Pflugers Archiv-European Journal of Physiology 447: 641–646. 14770311 10.1007/s00424-003-1068-1

[9] Gangolf M, Czerniecki J, Radermecker M, Detry O, Nisolle M, et al (2010) Thiamine Status in Humans and Content of Phosphorylated Thiamine Derivatives in Biopsies and Cultured Cells. PLoS ONE 5: 13. 10.1371/journal.pone.0013616 PMC2963613 21049048

[10] Lie MA, Celik L, Jorgensen KA, Schiott B (2005) Cofactor activation and substrate binding in pyruvate decarboxylase. Insights into the reaction mechanism from molecular dynamics simulations. Biochemistry 44: 14792–14806. 16274227 10.1021/bi051134y

[11] Navarro D, Zwingmann C, Butterworth RF (2008) Impaired oxidation of branched-chain amino acids in the medial thalamus of thiamine-deficient rats. Metabolic Brain Disease 23: 445–455. 18773288 10.1007/s11011-008-9105-6

[12] Agyei-Owusu K, Leeper FJ (2009) Thiamin diphosphate in biological chemistry: analogues of thiamin diphosphate in studies of enzymes and riboswitches. Febs Journal 276: 2905–2916. 19490097 10.1111/j.1742-4658.2009.07018.x

[13] Bettendorff L, Wirtzfeld B, Makarchikov AF, Mazzucchelli G, Frederich M, et al (2007) Discovery of a natural thiamine adenine nucleotide. Nature Chemical Biology 3: 211–212. 17334376 10.1038/nchembio867

[14] Frederich M, Delvaux D, Gigliobianco T, Gangolf M, Dive G, et al (2009) Thiaminylated adenine nucleotides. Chemical synthesis, structural characterization and natural occurrence. Febs Journal 276: 3256–3268. 19438713 10.1111/j.1742-4658.2009.07040.x

[15] Gigliobianco T, Lakaye B, Wins P, El Moualij B, Zorzi W, et al (2010) Adenosine thiamine triphosphate accumulates in Escherichia coli cells in response to specific conditions of metabolic stress. Bmc Microbiology 10: 12. 20492686 10.1186/1471-2180-10-148 PMC2881022

[16] Sriram K, Manzanares W, Joseph K (2012) Thiamine in Nutrition Therapy. Nutrition in Clinical Practice 27: 41–50. 22223666 10.1177/0884533611426149

[17] Gibson GE, Blass JP (2007) Thiamine-dependent processes and treatment strategies in neurodegeneration. Antioxidants & Redox Signaling 9: 1605–1619. 17685850 10.1089/ars.2007.1766

[18] Pan X, Gong N, Zhao J, Yu Z, Gu F, et al (2010) Powerful beneficial effects of benfotiamine on cognitive impairment and beta-amyloid deposition in amyloid precursor protein/presenilin-1 transgenic mice. Brain 133: 1342–1351. 20385653 10.1093/brain/awq069

[19] Tylicki A, Siemieniuk M (2011) Thiamine and its derivatives in the regulation of cell metabolism. Postepy Higieny I Medycyny Doswiadczalnej 65: 23. 10.5604/17322693.951633 21734329

[20] Loew D (1996) Pharmacokinetics of thiamine derivatives especially of benfotiamine. International Journal of Clinical Pharmacology and Therapeutics 34: 47–50. 8929745

[21] Greb A, Bitsch R (1998) Comparative bioavailability of various thiamine derivatives after oral administration. International Journal of Clinical Pharmacology and Therapeutics 36: 216–221. 9587048

[22] Volvert ML, Seyen S, Piette M, Evrard B, Gangolf M, et al (2008) Benfotiamine, a synthetic S-acyl thiamine derivative, has different mechanisms of action and a different pharmacological profile than lipid-soluble thiamine disulfide derivatives. BMC Pharmacol 8: 10. 18549472 10.1186/1471-2210-8-10 PMC2435522

[23] Aguilar F, Charrondiere UR, Dusemund B, Galtier P, Gilbert J, et al (2008) Scientific Opinion: Benfotiamine, thiamine monophosphate chloride and thiamine pyrophosphate chloride, as sources of vitamin B1 added for nutritional purposes to food supplements. EFSA Journal 864: 1–31.

[24] Franca DS, Souza AL, Almeida KR, Dolabella SS, Martinelli C, et al (2001) B vitamins induce an antinociceptive effect in the acetic acid and formaldehyde models of nociception in mice. Eur J Pharmacol 421: 157–164. 11516431 10.1016/s0014-2999(01)01038-x

[25] Song XS, Huang ZJ, Song XJ (2009) Thiamine suppresses thermal hyperalgesia, inhibits hyperexcitability, and lessens alterations of sodium currents in injured, dorsal root ganglion neurons in rats. Anesthesiology 110: 387–400. 19194165 10.1097/ALN.0b013e3181942f1e

[26] Moallem SA, Hosseinzadeh H, Farahi S (2008) A study of acute and chronic anti-nociceptive and anti-inflammatory effects of thiamine in mice. Iran Biomed J 12: 173–178. 18762821

[27] Balakumar P, Rohilla A, Krishan P, Solairaj P, Thangathirupathi A (2010) The multifaceted therapeutic potential of benfotiamine. Pharmacol Res 61: 482–488. 20188835 10.1016/j.phrs.2010.02.008

[28] Sanchez-Ramirez GM, Caram-Salas NL, Rocha-Gonzalez HI, Vidal-Cantu GC, Medina-Santillan R, et al (2006) Benfotiamine relieves inflammatory and neuropathic pain in rats. Eur J Pharmacol 530: 48–53. 16359659 10.1016/j.ejphar.2005.11.016

[29] Wang ZB, Gan Q, Rupert RL, Zeng YM, Song XJ (2005) Thiamine, pyridoxine, cyanocobalamin and their combination inhibit thermal, but not mechanical hyperalgesia in rats with primary sensory neuron injury. Pain 114: 266–277. 15733653 10.1016/j.pain.2004.12.027

[30] Winkler G, Pal B, Nagybeganyi E, Ory I, Porochnavec M, et al (1999) Effectiveness of different benfotiamine dosage regimens in the treatment of painful diabetic neuropathy. Arzneimittelforschung 49: 220–224. 10219465 10.1055/s-0031-1300405

[31] Stracke H, Gaus W, Achenbach U, Federlin K, Bretzel RG (2008) Benfotiamine in diabetic polyneuropathy (BENDIP): results of a randomised, double blind, placebo-controlled clinical study. Exp Clin Endocrinol Diabetes 116: 600–605. 18473286 10.1055/s-2008-1065351

[32] Nikolic A, Kacar A, Lavrnic D, Basta I, Apostolski S (2009) [The effect of benfothiamine in the therapy of diabetic polyneuropathy]. Srp Arh Celok Lek 137: 594–600. 20069914 10.2298/sarh0912594n

[33] Zylka MJ, Sowa NA, Taylor-Blake B, Twomey MA, Herrala A, et al (2008) Prostatic acid phosphatase is an ectonucleotidase and suppresses pain by generating adenosine. Neuron 60: 111–122. 18940592 10.1016/j.neuron.2008.08.024 PMC2629077

[34] Knyihar-Csillik E, Bezzegh A, Boti S, Csillik B (1986) Thiamine monophosphatase: a genuine marker for transganglionic regulation of primary sensory neurons. J Histochem Cytochem 34: 363–371. 3005391 10.1177/34.3.3005391

[35] Wada T, Takagi H, Minakami H, Hamanaka W, Okamoto K, et al (1961) A new thiamine derivative, S-benzoylthiamine O-monophosphate. Science 134: 195–196. 13782394 10.1126/science.134.3473.195

[36] Fairbanks CA (2003) Spinal delivery of analgesics in experimental models of pain and analgesia. Adv Drug Deliv Rev 55: 1007–1041. 12935942 10.1016/s0169-409x(03)00101-7

[37] Shields SD, Eckert WA, 3rd, Basbaum AI (2003) Spared nerve injury model of neuropathic pain in the mouse: a behavioral and anatomic analysis. J Pain 4: 465–470. 14622667 10.1067/s1526-5900(03)00781-8

[38] Lu J, Frank EL (2008) Rapid HPLC measurement of thiamine and its phosphate esters in whole blood. Clin Chem 54: 901–906. 18356241 10.1373/clinchem.2007.099077

[39] Yamazaki M (1968) Studies on the absorption of S-benzoylthiamine O-monophosphate: (I) Metabolism in tissue homogenates. Vitamins 38: 12–20.

[40] Quintero IB, Araujo CL, Pulkka AE, Wirkkala RS, Herrala AM, et al (2007) Prostatic acid phosphatase is not a prostate specific target. Cancer Res 67: 6549–6554. 17638863 10.1158/0008-5472.CAN-07-1651

[41] Ang CD, Alviar MJ, Dans AL, Bautista-Velez GG, Villaruz-Sulit MV, et al.. (2008) Vitamin B for treating peripheral neuropathy. Cochrane Database Syst Rev: CD004573. 10.1002/14651858.CD004573.pub3 PMC12373429 18646107

[42] Rindi G, Ricci V, Gastaldi G, Patrini C (1995) Intestinal alkaline phosphatase can transphosphorylate thiamin to thiamin monophosphate during intestinal transport in the rat. Archives of Physiology and Biochemistry 103: 33–38. 8574774 10.3109/13813459509007560

[43] Mihara Y, Utagawa T, Yamada H, Asano Y (2000) Phosphorylation of nucleosides by the mutated acid phosphatase from Morganella morganii. Applied and Environmental Microbiology 66: 2811–2816. 10877772 10.1128/aem.66.7.2811-2816.2000 PMC92077

[44] Mihara Y, Utagawa T, Yamada H, Asano Y (2001) Acid phosphatase/phosphotransferases from enteric bacteria. Journal of Bioscience and Bioengineering 92: 50–54. 16233057 10.1263/jbb.92.50

[45] Buchwald SL, Saini MS, Knowles JR, Vanetten RL (1984) Stereochemical course of phospho group transfer by human prostatic acid phosphatase. Journal of Biological Chemistry 259: 2208–2213. 6698963

[46] Petrov SA (1992) Studies of thiamine metabolism in organs and tissues of white mice in vivo and in vitro. Fiziologicheskii Zhurnal 38: 79–85. 1568503

[47] Lazareno S, Dolezal V, Popham A, Birdsall NJM (2004) Thiochrome enhances acetylcholine affinity at muscarinic M-4 receptors: Receptor subtype selectivity via cooperativity rather than affinity. Molecular Pharmacology 65: 257–266. 14722259 10.1124/mol.65.1.257

[48] Gomeza J, Zhang L, Kostenis E, Felder CC, Bymaster FP, et al (2001) Generation and pharmacological analysis of M-2 and M-4 muscarinic receptor knockout mice. Life Sciences 68: 2457–2466. 11392613 10.1016/s0024-3205(01)01039-6

[49] Cai YQ, Chen SR, Han HD, Sood AK, Lopez-Berestein G, et al (2009) Role of M2, M3, and M4 muscarinic receptor subtypes in the spinal cholinergic control of nociception revealed using siRNA in rats. J Neurochem 111: 1000–1010. 19780895 10.1111/j.1471-4159.2009.06396.x PMC4435670

[50] Hammes HP, Du X, Edelstein D, Taguchi T, Matsumura T, et al (2003) Benfotiamine blocks three major pathways of hyperglycemic damage and prevents experimental diabetic retinopathy. Nat Med 9: 294–299. 12592403 10.1038/nm834

